# COVID-19–Induced Flare of Hereditary Angioedema in a Twelve-Year-Old Female Patient

**DOI:** 10.7759/cureus.89210

**Published:** 2025-08-01

**Authors:** Aman Kalkat, Irfan Amir, Sarah Azzi, Gabrielle Tan, Robert Hostoffer

**Affiliations:** 1 Allergy and Immunology, Case Western Reserve University/University Hospitals Cleveland Medical Center Program, Cleveland, USA; 2 Internal Medicine, Case Western Reserve University/University Hospitals Cleveland Medical Center Program, Cleveland, USA; 3 Allergy and Immunology, Lake Erie College of Osteopathic Medicine, Erie, USA

**Keywords:** angioedema flare, bradykinin pathway, c1 esterase inhibitor deficiency, covid-19, hereditary angioedema, hereditary angioedema type 1, pediatric hereditary angioedema, sars-cov-2

## Abstract

Hereditary angioedema (HAE) is a rare disorder characterized by recurrent episodes of angioedema, most often due to a deficiency or dysfunction of C1 esterase inhibitor. This deficiency leads to an accumulation of bradykinin, a pro-inflammatory peptide that increases vascular permeability and causes localized swelling. Although some HAE flares occur spontaneously, known triggers include trauma, stress, and infection. Clinical manifestations typically involve swelling of the skin, gastrointestinal tract, and upper airway.

SARS-CoV-2, the virus responsible for COVID-19, enters host cells via angiotensin-converting enzyme 2 (ACE2). Viral binding and subsequent ACE2 depletion impair bradykinin degradation, leading to increased bradykinin levels, a mechanism that mirrors the pathophysiology of HAE. This shared pathway may contribute to HAE exacerbations during COVID-19 infections. We report the case of a pediatric patient with known HAE who experienced a disease flare triggered by COVID-19. While bradykinin-driven swelling in adult HAE patients with COVID-19 has been documented, pediatric reports are exceedingly rare. To our knowledge, this is the first published case detailing the symptom progression and treatment timeline of a confirmed pediatric HAE patient following SARS-CoV-2 infection. This case aims to raise awareness of COVID-19 as a potential trigger for HAE flares in children and emphasizes the need for healthcare providers to educate families of pediatric HAE patients about flare management and preparedness in the context of COVID-19, especially given its continued global circulation.

## Introduction

Hereditary angioedema (HAE) is a disorder characterized by recurrent episodes of angioedema, most commonly caused by a deficiency of C1 esterase inhibitor. This deficiency results in elevated levels of bradykinin, a pro-inflammatory peptide that promotes vasodilation and increases vascular permeability. Commonly affected areas include the skin, gastrointestinal tract, and upper airway. Flares can be spontaneous or triggered by pressure, trauma, stress, or infection [1]. With the growing prevalence of COVID-19, concerns have emerged regarding the potential interaction between HAE and SARS-CoV-2 infection, given the overlapping pathophysiological mechanisms.

SARS-CoV-2, the virus responsible for COVID-19, enters host cells via the angiotensin-converting enzyme 2 (ACE2) receptor. Viral binding leads to ACE2 depletion, impairing bradykinin degradation and resulting in elevated bradykinin levels, similar to the mechanism seen in HAE [2].

Patients with HAE are hypothesized to be at higher risk for COVID-19-related complications. In a study by Veronez et al., individuals with C1 esterase inhibitor deficiency who were receiving subcutaneous replacement therapy reported fewer COVID-19 cases compared to those not on treatment. This finding supports the idea that elevated uninhibited bradykinin may contribute to the pathogenesis of both HAE and COVID-19 [3]. We present the clinical course of a pediatric patient with type 1 HAE whose flare was triggered by a confirmed COVID-19 infection. Although one prior pediatric case has been reported, it lacks publicly available details regarding symptom onset, prophylaxis timing, and treatment outcomes [4]. This case report provides a documented example of COVID-19-induced HAE flare in a pediatric patient, including full clinical context and management details.

## Case presentation

The patient was initially evaluated at eight years of age for recurrent monthly episodes of bilateral arm and intestinal wall angioedema, which had been occurring since early childhood. At that time, her symptoms raised concern for structural causes of acute abdominal pain. Recurrent intense abdominal pain in children may mimic conditions such as appendicitis, cholecystitis, volvulus, intussusception, or bowel ischemia. Historically, many patients with HAE have undergone unnecessary surgical procedures due to its presentation resembling an acute abdomen. In this case, the simultaneous occurrence of arm and abdominal angioedema suggested a systemic etiology. The absence of urticaria pointed away from an allergic cause, a suspicion further supported by a normal serum tryptase level. The symptom resolution pattern also aligned with HAE, and abdominal CT imaging during an acute episode may have demonstrated bowel wall edema. Laboratory evaluation revealed reduced C1 esterase inhibitor function and antigen levels, along with decreased C4 levels. C3 and tryptase levels were within normal limits (Table 1). These findings confirmed a diagnosis of type 1 HAE. The patient was started on C1 esterase inhibitor replacement therapy three to four times weekly, which led to a reduction in flare frequency. Due to challenging peripheral venous access, a central venous port was placed to facilitate treatment. Over time, the dosing was titrated, and the patient was successfully maintained on 1000 IU of C1 esterase inhibitor twice weekly.

**Table 1:** Initial laboratory values at age 8

Test	Result	Reference range	Interpretation
C1 esterase inhibitor (function)	44%	>70%	Decreased
C1 esterase inhibitor (antigen)	5 mg/dL	19–37 mg/dL	Decreased
C4	3 mg/dL	12–42 mg/dL	Decreased
C3	84 mg/dL	80–178 mg/dL	Normal
Tryptase	4.4 ng/mL	<15 ng/mL	Normal

**Figure 1:**
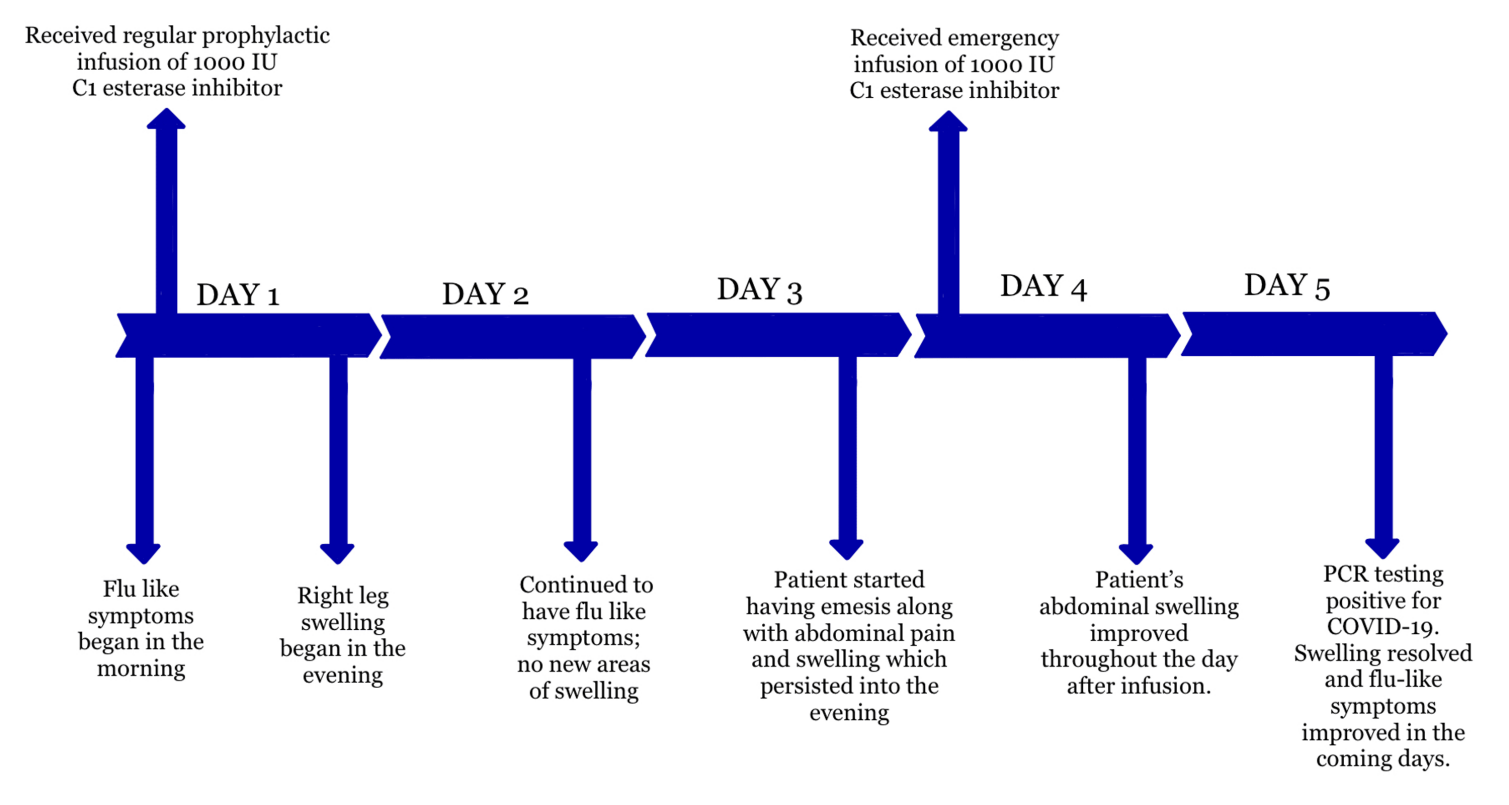
Timeline of the patient’s presentation, interventions, and COVID-19 diagnosis Occurrences of both prophylactic and emergency C1 esterase inhibitor administration are shown above the timeline. The patient's symptoms during the five-day course are shown below the timeline.

The patient remained stable without HAE flares on her maintenance regimen for several years until she contracted COVID-19 at 12 years of age. On the first day of illness, she developed flu-like symptoms including body aches, headache, fever, and nausea. Her family recorded a fever of 101.0°F at home. That evening, she experienced right leg edema lasting through the night, despite having received her scheduled prophylactic infusion of C1 esterase inhibitor (1000 IU) earlier that day. During the flare, the patient had a low-grade fever but no hypotension or respiratory distress. The following day, no new areas of swelling developed, although flu-like symptoms persisted. On the third day, she experienced multiple episodes of emesis accompanied by severe abdominal pain and distension. According to her mother, the abdominal swelling was more severe than in any of her previous episodes. On day four, she was given an emergency dose of C1 esterase inhibitor (1000 IU), after which her symptoms began to improve within hours. Complete resolution of swelling occurred within approximately 4-6 hours, consistent with expected response times to rescue therapy in pediatric HAE patients. The next day, she visited her pediatrician and tested positive for COVID-19 via PCR. She recovered well from the infection and did not experience additional episodes of acute angioedema during that week (Figure 1). Her flu-like symptoms gradually resolved over the following days, and she resumed her maintenance regimen of C1 esterase inhibitor at 1000 IU twice weekly.

## Discussion

The pathophysiological interaction between COVID-19 and HAE is largely driven by elevated bradykinin levels, which contribute to inflammation, vasodilation, and potential lung injury [5]. COVID-19 infection disrupts the renin-angiotensin system by downregulating ACE2, an enzyme responsible for degrading bradykinin. As a result, bradykinin accumulates, leading to increased vascular permeability and tissue angioedema [2].

Xu et al. examined the interaction between COVID-19 and HAE and proposed that, due to shared physiological pathways, comorbidity with HAE may increase the risk of COVID-19 progression and lead to worse outcomes. Conversely, COVID-19 could potentially exacerbate pre-existing HAE or even trigger symptom onset in asymptomatic carriers of HAE-related mutations [5]. Additionally, Rios Rodriguez et al. reported a case of acquired angioedema following COVID-19 infection in a 37-year-old man, suggesting that post-COVID-19 angioedema may be linked to long-term angioedema manifestations [6]. 

In addition to the direct effects of COVID-19 infection, stress related to the pandemic may have also contributed to increased HAE flares. Christiansen et al. surveyed patients with HAE and found a rise in attack frequency during the COVID-19 pandemic, even among those without confirmed infection [7]. These findings suggest that psychological stressors associated with COVID-19 may have exacerbated the flare in our patient [7].

Olivares et al. studied 56 patients over the age of 10 with a history of HAE who contracted COVID-19, and 24 of them experienced an acute angioedema attack [8]. While two patients in the 10-20 age range were included, it is unclear whether they developed a flare during the course of their infection [8]. The study did not provide a detailed clinical timeline of symptom onset, severity, or treatment, limiting its relevance to pediatric cases. Furthermore, no specific information was given regarding the progression or resolution of symptoms in any of the patients.

In another study, Farinha et al. evaluated 34 adult patients with HAE, of whom 16 contracted COVID-19 [9]. Among these 16 patients, 25% experienced angioedema attacks during their infection, and 43.8% had attacks during the three-month convalescence period, despite all having mild COVID-19, with most not requiring hospitalization [9]. Our case is unique in that it involves a pediatric patient who experienced a severe angioedema flare requiring emergency intervention despite being on regular prophylaxis. Similarly, Szilágyi et al. assessed the effects of COVID-19 in patients with HAE types 1 and 2, finding that 25% experienced attacks during the pandemic [10]. However, their study did not include pediatric patients, further highlighting the importance of our case in expanding the current understanding of HAE in children with COVID-19. 

Our patient developed angioedema despite having received prophylactic C1 esterase inhibitor therapy just 48 hours prior to symptom onset and required an additional emergency dose for symptom resolution. This underscores the potential for COVID-19 to trigger breakthrough attacks in pediatric HAE patients, possibly overwhelming the protection provided by standard prophylaxis. The scarcity of similar pediatric cases in the literature further emphasizes the importance of reporting this case to better understand the impact of COVID-19 on children with HAE.

Vaccination against COVID-19 may offer an added layer of protection for patients with HAE. A study by Özdemir et al. found that among HAE patients who received multiple doses of different COVID-19 vaccines, there were no significant increases in the frequency of angioedema attacks [11]. These findings support the safety of COVID-19 vaccination in HAE patients and reinforce its potential value, especially given the increased risk of severe flares associated with COVID-19 infection.

## Conclusions

Healthcare providers should be aware of the potential for HAE flares in pediatric patients with concurrent COVID-19 infection, even among those receiving regular prophylactic C1 esterase inhibitor therapy. By detailing the clinical course and management of a pediatric patient with COVID-19-induced HAE, we aim to enhance clinicians’ understanding of this rare presentation and provide insight into strategies for effective management in children. While several studies have explored the relationship between HAE and COVID-19, most have focused exclusively on adults, leaving a significant gap in pediatric data. This case highlights the importance of recognizing COVID-19 as a potential trigger for HAE attacks in children, underscoring the need for close monitoring and timely intervention, regardless of prophylactic treatment status. Moreover, it draws attention to the lack of pediatric-focused research on this topic. Further studies are needed to better characterize the impact of COVID-19 on children with HAE and to develop tailored management approaches for this population. Increased clinical awareness and deeper understanding of this interaction may lead to earlier recognition and improved outcomes in affected pediatric patients.
